# The Relationship Between Generalised Joint Hypermobility and Autism Spectrum Disorder in Adults: A Large, Cross-Sectional, Case Control Comparison

**DOI:** 10.3389/fpsyt.2021.803334

**Published:** 2022-02-08

**Authors:** Martin R. Glans, Nils Thelin, Mats B. Humble, Marie Elwin, Susanne Bejerot

**Affiliations:** ^1^School of Medical Sciences, Örebro University, Örebro, Sweden; ^2^Division of Psychiatry, Linköping University Hospital, Linköping, Sweden; ^3^Faculty of Medicine and Health, University Health Care Research Centre, Örebro University, Örebro, Sweden; ^4^Department of Clinical Neuroscience, Karolinska Institutet (KI), Solna, Sweden

**Keywords:** comorbidity [MeSH], joint hypermobility, Ehlers-Danlos syndrome, biomarker, connective tissue, autism spectrum disorder (ASD), adults

## Abstract

Autism spectrum disorder (ASD) and generalised joint hypermobility (GJH) share a number of clinical manifestations including proprioceptive impairment, motor difficulties, sensory hypersensitivity, and autonomic dysfunction. Clinical observations suggest that GJH is overrepresented in ASD. However, there are currently few systematic studies available. Knowledge about comorbidities may unfold common aetiopathological pathways underlying the association and improve the clinical management. The aim of this large, cross-sectional comparative study is to evaluate the relationship between ASD and GJH in adults. Data on joint hypermobility, symptoms associated with both hypermobility spectrum disorders (HSD) and hypermobile Ehlers-Danlos syndrome (hEDS), lifetime psychiatric diagnoses, psychiatric rating scales for ASD and attention deficit hyperactivity disorder (ADHD), and socio-demographics was collected for 199 individuals with ASD and 419 non-ASD community controls. Logistic regression models adjusting for covariates (age, sex, ethnicity) revealed a significant relationship between ASD and GJH and between ASD and symptomatic GJH, with adjusted odds ratios of 3.1 (95% CI: 1.9, 5.2; *p* < 0.001) and 4.9 (95% CI: 2.6, 9.0; *p* < 0.001), respectively. However, the high prevalence of comorbid ADHD in the study sample reduces the generalizability of the results among individuals with ASD without comorbid ADHD. Possibly, an additional ADHD phenotype is the primary driver of the association between ASD and GJH. Furthermore, GJH with additional self-reported symptoms, suggestive of HSD/hEDS, showed a stronger association with ASD than did non-specified GJH, indicating that symptomatic GJH plays a greater role in the relationship than non-specified GJH does. Therefore, the current study underscores the need of careful sample subclassifications. ASD with GJH may represent a novel subgroup of ASD in terms of aetiopathology and clinical presentation. Future research should elucidate the aetiological factors behind the association between ASD and GJH and evaluate how the comorbidity of GJH affects ASD outcomes.

## Introduction

Autism Spectrum Disorder (ASD) is comprised of a group of conditions characterised by persistent difficulties in social interaction and communication, as well as restricted and repetitive patterns of behaviour, interests, or activities (1). People with ASD tend to be easily stressed, overwhelmed, and lonely. The vast majority are bullied in school (2) and, regardless of cognitive level, can expect hardships in finding a suitable job in adulthood (3).

ASD is associated with several comorbid psychiatric and physical conditions (4–6). However, the reported prevalence rates vary widely between studies, likely related to the clinical and pathogenetic heterogeneity of ASD (4). The 2013 update (5th edition) of the Diagnostic and Statistical Manual of Mental Disorders (DSM-5) permitted a co-occurring ADHD diagnosis with ASD (1). One recent meta-analysis estimated the lifetime prevalence of ADHD amongst patients with ASD to be approximately 40% (7), however, depression, obsessive-compulsive disorder and anxiety are also common (6), in addition to gender dysphoria (8). Physical comorbidities e.g., sleep related problems, epilepsy, sensory impairments, atopy, autoimmune conditions, clumsiness, and obesity are also frequently reported (5). Due to the dearth of studies and/or studies with small or highly selective samples, the evidence is limited for other comorbidities (5). Knowledge about comorbidities is important, given that this may provide clues to underlying aetiological factors (4). Furthermore, comorbidities may substantially impair the quality of life in already severely distressed people. Therefore, the recognition of comorbidities is critical for treatment and to improve understanding and outcomes. Generalised joint hypermobility (GJH) is one such condition that has recently been proposed as a neglected comorbidity in ASD (9).

GJH is defined as the ability to move several synovial joints beyond normal limits and occurs with relative frequency in the general population, with a reported prevalence around 10–20%. It is influenced by age, sex, and ethnicity (10, 11). GJH is frequently asymptomatic but may also be associated with a broad range of musculoskeletal and extra-musculoskeletal manifestations. Furthermore, GJH is the hallmark of various hereditary connective tissue disorders, including Ehlers Danlos syndromes (EDS). The hypermobile Ehlers-Danlos syndrome (hEDS) is characterised by GJH combined with systemic manifestations of a connective tissue disorder [e.g., velvety skin, skin hyperelasticity, and recurrent or multiple abdominal hernia(s)] and musculoskeletal complications (e.g., limb pain, chronic widespread pain, and recurrent dislocated joints) (12). If the diagnostic criteria for hEDS are not fully met, the condition will frequently be classified as hypermobility spectrum disorder (HSD), which bridges the gap between asymptomatic GJH and hEDS (10). To date, not much is known about the underlying mechanisms for GJH. For many of the EDS subtypes, pathogenic variants of genes that encode collagen and collagen-related structures have been identified. However, for the most common forms of symptomatic GJH, namely hEDS and HSD, the genetic basis remains unknown (10, 12).

Interestingly, ASD and GJH share a number of clinical manifestations, including proprioceptive impairment, gross motor difficulties, sensory hypersensitivity, and autonomic dysfunction (13, 14). Additionally, ASD as well as hEDS/HSD are highly hereditary (10, 15) and an aetiological overlap between the two conditions has been suggested (9, 14). Yet, despite the growing body of literature in the area, few systematic studies are available. To our knowledge, only a handful of case reports (13, 16–20), one cross-sectional study of children with ASD (21), and one case-control study in children (22) have reported an association between ASD and GJH. In the case-control study, the sample size was quite small and the lack of age norms for joint mobility prevented using standardised assessments for GJH, limiting the generalisability of the findings. Additionally, two registry-based studies demonstrated that having an ASD diagnosis was more common in those with HSD/hEDS compared to the general population (23, 24). However, since HSD/hEDS and ASD are both spectrum conditions, typically treated by two separate fields of medicine, they and their combination are likely to be underdiagnosed. Moreover, GJH/HSD/hEDS is strongly sex-skewed towards females (10, 11), and females (particularly those with IQ in the average/above average range) are underdiagnosed with ASD (25). The two registry studies included HSD/hEDS patients from hospital-based settings only. Thus, the included cases may have demonstrated a higher comorbidity burden than the overall HSD/hEDS population, with a risk of inflating the association estimates between GJH and psychiatric disorders. Therefore, registry-based studies, which rely on confirmed diagnoses, may be liable of e.g., referral bias and surveillance bias. Consequently, the current study was designed as a large case-control study, which measured GJH status in individuals diagnosed with ASD and community controls, with a sufficient sample size allowing for adjusted analyses for recognised influencers of GJH (age, sex, and country of origin).

### Aims of the Study

The primary aim of the current study was to assess the relationship between ASD and GJH. The secondary aim was to also evaluate the hypothesised association in symptomatic GJH.

## Materials and Methods

### Participants and Enrolment

The current study is a cross-sectional case-control comparison carried out in Sweden between May 2015 and February 2020. A total of 618 participants, 199 patients with ASD and 419 non-ASD community controls, were included in the study. The rule of event per variable with a formula of *n* = 100 +50*i*, in which *i* refers to number of independent variables in the final model, was used, and as such, a minimum sample size of 300 was targeted. The recommended sample size was exceeded to enable additional analyses on the material.

The study participants who had been diagnosed with ASD were recruited from outpatient clinics for individuals with adult ASD or ADHD, located in Stockholm (*n* = 161) and Linköping (*n* = 30) and from inpatient psychiatric facilities located in Stockholm (*n* = 8). The study control participants were utilised in a previous study on GJH and ADHD (26). They were recruited from Stockholm from a university campus (*n* = 232), as patients and accompanying persons attending a community health centre (*n* = 110), and from health-care staff and other professionals from various workplaces (*n* = 77). All participants were provided information on the study goals to gather information on psychiatric characteristics and joint mobility and on the anonymity of the study. Participants were asked to complete a survey form, be physically examined for joint hypermobility, and respond to questions on psychiatric diagnoses. The physical examination was performed by a trained physician who was blinded to the results of the 5PQ (Martin Glans in Stockholm and Nils Thelin in Linköping). A goniometer measured the fifth finger, elbow, and knee while the examinee was in the standing position. The participants did not warm-up prior to the examination and any physical condition interfering with the examination was noted.

Inclusion criteria were fluency in Swedish and being in the age range of 18–65 years old. Exclusion criteria were missing data for age, sex, country of origin, or psychiatric diagnoses. Among the presumptive control participants, those who presented with ASD were excluded. Additional exclusion criteria depended on the analysis as follows: for the Beighton Scoring System (BSS), missing data or a physical condition affecting the cut-off score for GJH; for the 5PQ, missing data; for symptomatic GJH, missing data on the four items regarding musculoskeletal symptoms and skin abnormalities; for the AQ-10, missing data; and for the ASRS analyses, any more than one missing item from each subscale. In such cases when one item was missing from any ASRS subscale, the mean substitution method was used for imputation.

#### Ethics Statement

All procedures that contributed to the current study were conducted consistent with the guidelines set forth by each national and institutional committee, as dictated by the Helsinki Declaration of 1975 (revised in 2008) and were approved by the regional Ethics Review Board of Stockholm (approval numbers 2014/1742-31, 2017/1688-31, and 2017/2140-32). All participants provided signed informed consent.

### Assessments

The survey form contained demographic questions, the 5PQ, items concerning musculoskeletal symptoms and skin abnormalities, psychiatric rating scales, and a self-report of lifetime history of psychiatric diagnoses. Completion of the survey was followed by a physical examination.

#### Demographics

Age (in years), sex (binary male/female), country of origin, employment status, and educational level were included for demographic characteristics. Country of origin was designated by a question on either parent being born outside of Nordic countries, with a follow up question as to where each respective parent was born, if so indicated.

#### Psychiatric Diagnoses

Information about psychiatric diagnoses relied on self-reporting in the survey followed by questions about lifetime psychiatric diagnoses by the assessors (Martin Glans and Nils Thelin). The survey form included specific questions on a past (lifetime) or current diagnosis with autism, atypical autism, or Asperger syndrome, ADHD/ADD, depression, anxiety disorder (such as social phobia or panic disorder), or other psychiatric disorders. If the subject answered yes for other psychiatric disorders, the assessor would probe to ascertain which disorder the participant had been diagnosed with.

#### Psychiatric Rating Scales

The psychiatric rating scales were included in the survey for descriptive purposes and to enable comparisons of psychiatric characteristics between groups. The WHO Adult ADHD Self-Report Scale (ASRS) is a widely used, 18 item screening instrument for assessing adult ADHD, including two subscales, one on hyperactivity/impulsivity (Hy/Imp) and one on inattention (Inatt). Each item is rated on a Likert-type scale from 0 to 4 (0 = never, 1 = rarely, 2 = sometimes, 3 = often, and 4 = very often), yielding a total range of 0–72, with each subscale ranging from 0–36. A significant correlation (*r* = 0.43) between total scores and clinical symptom severity has been demonstrated, supporting the use of continuous scoring (27).

The Autism Spectrum Quotient-10 items (AQ-10) is a brief screening instrument for examining autistic symptoms (28). Consistent with the idea of autistic traits as a dimension, and to retain information about trait values, a continuous scoring method of 0–3 for each item was used, yielding a total range of 0–30 (29).

#### Generalised Joint Hypermobility

To date, there is no gold standard test for classifying GJH, although the Beighton Scoring System (BSS) is often used as such (30). The current study used two independent assessment methods for GJH: a physical examination following the BSS and the five-part questionnaire (5PQ), a self-reported screening tool for hypermobility. The BSS is a nine-point evaluation of four bilateral and one unilateral joint. Each joint identified with hypermobility is counted as 1 point, for a total score ranging from 0 to 9 (31). Age-dependent cut-off scores of ≥ 5 for individuals aged 18–50 years and ≥ 4 for individuals aged > 50 years were used in the current study, according to the 2017 updated criteria (12). However, to facilitate comparability, prevalence rates of GJH with a cut-off ≥ 4 /9 was presented for all ages. The 5PQ covers five questions, with each positive answer scoring one point. A cut-off value of ≥ 2 was applied for the 5PQ (32, 33).

#### Symptomatic GJH

GJH is an umbrella term used to describe both asymptomatic and symptomatic GJH, hereby referred to as non-specified GJH. To identify a symptomatic subgroup as a clinical clue for connective tissue disease (e.g., suggestive of HSD/hEDS) four self-reported yes/no questions were used: “Do you often have pain in your back or in your joints?”; “Do you have hyper-elastic skin?”; “Do you have velvety skin?”; and “As a child or teenager, did your kneecap or shoulder dislocate on more than one occasion?”. Responses were then pooled with the BSS and 5PQ results to categorise participants with symptomatic GJH as either with symptomatic GJH-BSS or symptomatic GJH-5PQ, yielding four dependent variables (GJH-BSS, symptomatic-GHJ-BSS, GJH-5PQ, and symptomatic GJH-5PQ). Symptomatic GJH required at least one confirmatory response for one of the four additional items, plus GJH as defined by the BSS or the 5PQ, respectively.

### Analyses

A Student's t-test was used to assess continuous variables, while a Chi-square test, or Fisher's exact test when an expected value of a cell was < 5, was used for categorical variables for descriptive analyses and to examine group differences. Four logistic regression models were used to assess the association between ASD and GJH. The predictive variable was a diagnosis of ASD, with sex (female/male), age (years), and country of origin entered as covariates. Country of origin was dichotomised as “No parent born outside of the Nordic countries” or “At least one parent born outside the Nordic countries”. The four dependent variables were non-specified GJH as defined by the BSS, non-specified GJH as defined by the 5PQ, symptomatic GJH as defined by the BSS, and symptomatic GJH as defined by the 5PQ. The linearity assumption between the continuous independent variable (age) and the log odds of the dependent variable was tested using the Box-Tidwell Test. Multicollinearity was tested by determining correlation coefficients between predictor variables and employing a threshold of r > 0.7, as well as by determining the variance inflation factor and employing a threshold of 2.5. The robustness of the logistic regression models was assessed using a series of sensitivity analyses: (1) increasing the details on coding for country of origin, with separate variables based on the specific continent where each parent was born; (2) inclusion of the variable “any anxiety disorder”; and (3) exclusion of the variable “age” in the regression models. Finally, a subgroup analysis on patients with ASD without comorbid ADHD was run. All statistical analyses were conducted using IBM SPSS Statistics for Macintosh (version 27) and a two-sided *p* < *0.0*5 was used to determine statistical significance.

## Results

### Characteristics of the Study Population

**Figure 1:**
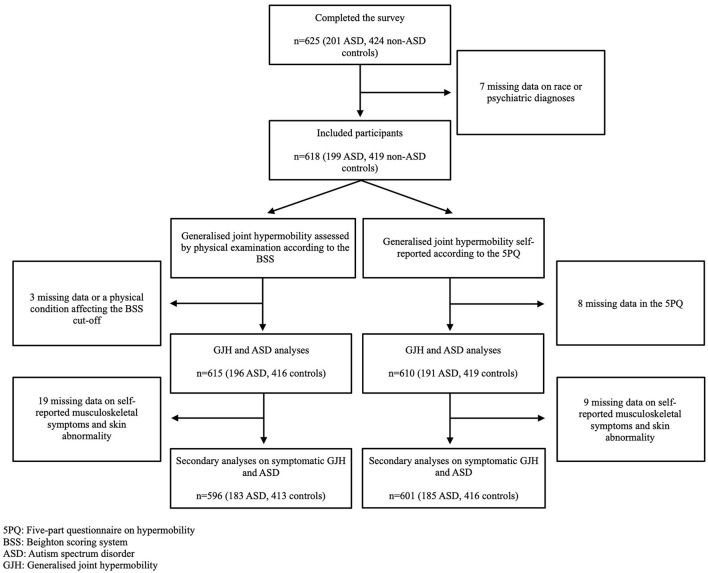
Flow of participants through the study.

A total of 618 participants, 199 with ASD and 419 non-ASD community controls, met the overall inclusion and exclusion criteria for the study, with additional exclusions made for specific analyses (Figure 1). The demographic and clinical characteristics of participants with ASD and the controls are shown in Table 1. Musculoskeletal symptoms and skin abnormalities were more frequent in those with ASD compared to non-ASD controls (Table 1). Additionally, participants with ASD comorbid with ADHD scored significantly higher on the ASRS total- and subscale scores compared to participants with ASD without comorbid ADHD. There was no significant difference in AQ-10 score when comparing ASD participants with and without a comorbid ADHD (Supplementary Table S1).

**Table 1:** Characteristics of the study population.

	**Group affiliation**		
	**ASD**	**Non-ASD controls**	
Variable	*N* = 199	*N* = 419	Test of difference
Demographics			
Female sex, n (%)	95 (47.7)	246 (58.7)	X^2^ = 6.57, *p =* 0.010
Age (yrs), (mean, SD)			
Women	33.2 (11.3)	32.5 (12.9)	t = −0.426, *p =* 0.671
Men	33.7 (12.2)	31.7 (12.1)	t = −1.30, *p =* 0.193
Ethnicity Nordic^a^, *n* (%)			
Women	74 (77.9)	185 (75.2)	X^2^ = 0.272, *p =* 0.602
Men	80 (76.9)	133 (76.9)	X^2^ = 0.000, *p =* 0.993
Employment status, n (%) (*n =* 137 v. 300)			
Employed or Student	62 (45.3)	299 (99.7)	X^2^ = 193.8, *p* < 0.001
Unemployed	75 (54.7)	1 (0.3)	
Highest completed education, *n* (%) (*n =* 141v. 408)			X^2^ = 119.9, *p* < 0.001
University ≥ 3 years	19 (13.5)	87 (21.3)	
University <3 years	16 (11.3)	23 (5.6)	
Upper Secondary school	58 (41.1)	268 (65.7)	
Vocational training	6 (4.3)	25 (6.1)	
Compulsory school	34 (24.1)	3 (0.7)	
Unfinished compulsory school	8 (5.7)	2 (0.5)	
Lifetime occurrence of psychiatric diagnoses^b^, *n* (%)			
Any (except ASD)	188 (94.5)	82 (19.6)	X^2^ = 307.7, *p* < 0.001
Depression	149 (74.9)	62 (14.8)	X^2^ = 216.6, *p* < 0.001
ADHD	138 (69.3)	3 (0.7)	X^2^ = 360.9,*p* < 0.001
Anxiety disorder	85 (42.7)	17 (4.1)	X^2^ = 146.3, *p* < 0.001
Exhaustion disorder^c^	13 (6.5)	7 (1.7)	X^2^ = 10.2, *p =* 0.001
Bipolar disorder	13 (6.5)	5 (1.2)	X^2^ = 13.6, *p* < 0.001
Specific learning disorder	12 (6.0)	4 (1.0)	X^2^ = 13.8, *p* < 0.001
Personality disorder	9 (4.5)	1 (0.2)	F, *p* < 0.001
PTSD	8 (4.0)	0 (0.0)	F, *p* < 0.001
Psychosis other^d^	8 (4.0)	0 (0.0)	F, *p* < 0.001
Eating disorder	4 (2.0)	5 (1.2)	F, *p =* 0.479
Intellectual disability	3 (1.5)	0 (0.0)	F, *p =* 0.033
Tourette syndrome	2 (1.0)	0 (0.0)	F, *p =* 0.103
Schizophrenia	2 (1.0)	1 (0.2)	F, *p =* 0.244
Substance use disorder	2 (1.0)	0 (0.0)	F, *p =* 0.103
Dissociative disorder	2 (1.0)	0 (0.0)	F, *p =* 0.103
Intermittent explosive disorder	2 (1.0)	0 (0.0)	F, *p =* 0.103
Psychiatric rating scales (mean, SD) (*n =* 178 v. 416)			
ASRS total score^e^			
Women	44.9 (11.4)	27.9 (10.1)	t = −13.0, *p* < 0.001
Men	40.5 (11.4)	29.1 (10.0)	t = −8.46, *p* < 0.001
ASRS Hyperactivity/Impulsivity subscale			
Women	19.7 (6.9)	13.2 (5.6)	t = −8.72, *p* < 0.001
Men	17.3 (6.6)	13.6 (5.8)	t = −4.70, *p* < 0.001
ASRS Inattention subscale			
Women	25.2 (6.1)	14.8 (5.6)	t = −14.6, *p* < 0.001
Men	23.2 (6.1)	15.4 (5.5)	t = −10.5, *p* < 0.001
Autism quotient abridged 10-item version^f^			
Women	19.4 (4.7)	9.4 (3.6)	t = −20.4,*p* < 0.001
Men	17.3 (4.7)	10.3 (3.7)	t = −13.2, *p* < 0.001
Musculoskeletal symptoms and skin abnormalities^g^, *n* (%) (*n =* 186 vs. 416)			
Any	141 (75.8)	214 (51.4)	X^2^ = 31.5, *p* < 0.001
Frequent pain in back or joints	126 (67.7)	167 (40.1)	X^2^ = 39.2, *p* < 0.001
Dislocated shoulder or kneecap ≥2	26 (14.0)	21 (5.0)	X^2^ = 14.2, *p* < 0.001
Hyperelastic skin	20 (10.8)	18 (4.3)	X^2^ = 8.97, *p =* 0.003
Velvety skin	47 (25.3)	59 (14.2)	X^2^ = 10.9, *p =* 0.001

*Abbreviations: ASD, autism spectrum disorder; ADHD, attention-deficit/hyperactivity disorder; ASRS, Adult ADHD Self Report Scale; PTSD, post-traumatic stress disorder; X^2^, Pearson Chi-squared test; t, Student t-test; F, Fisher's exact test*.

*Note: Comparisons are made between participants with ASD and non-ASD controls. Age analysed by Student t-test and categorical variables by Pearson Chi-squared test. Fisher's exact test was used when expected value of a cell was < 5. All p values are 2-sided. Autism spectrum disorder was an exclusion criteria for the control group. Missing data on a variable served as exclusion criteria, therefore the number of included participants differ between variables*.

a *Neither parent born outside of the Nordic countries*.

b *Lifetime occurrence of self-reported psychiatric diagnoses. Those with a reported prevalence lower than 1% are not shown*.

c *Exhaustion disorder was introduced as a medical diagnosis in Sweden by the Swedish National Board of Health and Welfare in 2010 and is equivalent to “burnout”*.

d *Unspecified psychosis not due to a substance or known physiological condition (n = 7), Substance-induced psychosis (n = 1)*.

e *Adult ADHD Self Report Scale; continuous scoring method (0–4 on each item, total score range 0–72)*.

f *Autism quotient abridged 10-item version, continuous scoring method (0–3 on each item, total score range 0–30)*.

g *Symptoms suggestive of symptomatic GJH (e.g. HSDs or h-EDS), were assessed by the four items; “Do you often have pain in your back or in your joints?”; “Do you have hyperelastic skin?”; “Do you have velvety skin?”, and “As a child or teenager, did your kneecap or shoulder dislocate on more than one occasion?”*.

### Prevalence Rates of GJH

Sex-stratified analyses comparing the prevalence rates of GJH between those with ASD and non-ASD controls are presented in Table 2. To facilitate comparability, the prevalence rates of GJH according to the non-age-dependent criteria of the BSS, which was the established recommendations prior to the update in 2017, are also presented. With a cut-off ≥ 4/9 on the BSS, the prevalence rates of GJH were 44.7 vs 24.0%, (χ2 = 12,2, *p* < 0.001) for women and 21.6 vs 7.6% (χ2 = 10.4, *p* < 0.001) for men, comparing participants with ASD to non-ASD controls and prevalence rates of symptomatic GJH were 37.5 vs 12.7% (χ2 = 2.8, *p* < 0.001) for women and 13.1 vs. 2.4% (Fisher's exact test *p* = 0.001) for men, also comparing participants with ASD to non-ASD controls.

**Table 2:** Prevalence of generalised joint hypermobility and symptomatic generalised joint hypermobility.

	**Group affiliation**		**Chi-Square tests**		**Risk estimate**	
	**ASD**	**Non-ASD**	**χ2** **(***df*** = 1)**	***P***	**OR**	**(95% CI)**
GJH as defined by the BSS^a^, *n* (%)						
Women	26 (28.0)	27 (11.0)	14.75	<0.001	3.15	(1.72-5.76)
Men	11 (10.7)	8 (4.7)	3.54	0.060	2.42	(0.940-6.24)
GJH as defined by the 5PQ^b^, *n* (%)						
Women	47 (51.1)	94 (38.2)	4.57	0.033	1.69	(1.04-2.74)
Men	32 (32.3)	36 (20.8)	4.45	0.035	1.82	(1.04-3.18)
Symptomatic^c^ GJH-BSS, *n* (%)						
Women	23 (26.1)	17 (7.0)	22.43	<0.001	4.73	(2.38-9.37)
Men	7 (7.4)	3 (1.8)	Fisher's	0.038	4.40	(1.11–17.4)
Symptomatic GJH-5PQ, *n* (%)						
Women	42 (47.2)	61 (25.0)	15.03	<0.001	2.68	(1.61–4.45)
Men	23 (24.0)	22 (12.8)	5.50	0.019	2.15	(1.12–4.11)

*Abbreviations: 5PQ, five-part questionnaire on hypermobility; ADHD, attention-deficit/hyperactivity disorder; BSS, Beighton scoring system; GJH, generalised joint hypermobility disorder; OR, odds ratio*.

*Note: Comparisons are made between participants with ASD and non-ASD controls and by sex. Difference between groups were analysed by Pearson Chi-square test. Fisher's exact test was used when expected value of a cell was < 5. All p values are 2-sided*.

a *GJH as defined by the Beighton scoring system; age-dependent cut-off score of ≥ 5/9 for individuals 18-50 years and ≥4/9 for individuals > 50 years*.

b *GJH as defined by the 5PQ; cut-off score ≥ 2/5*.

c *Symptomatic GJH-BSS and symptomatic GJH-5PQ were defined as GJH (as defined by the BSS and the 5PQ, respectively) combined with ≥1 out of 4 self-reported items: (1) back or joint pain, (2) dislocation of shoulder or patella more than once as a child or teenager, (3) skin hyperelasticity or (4) velvety skin*.

### Results of the Logistic Regression Analyses on the Association Between ASD and GJH

The linearity assumption was met for all analyses and there was no indication of multicollinearity (see Table 3). Sensitivity analyses are presented in the Supplementary Materials.

**Table 3:** Results of the logistic regression models on ASD diagnosis relationshi*p* with generalised joint hypermobility.

	**Unadjusted models**						**Adjusted models**					
	**B**	**SE**	**Wald**	***df***	***p***	**Unadjusted OR (95% CI)**	**B**	**SE**	**Wald**	***df***	***p***	**Adjusted OR (95% CI)**
Predictor												
GJH as defined by the BSS^a^												
ASD	0.929	0.254	13.4	1	*p* < 0.001	2.53 (1.54–4.17)	1.13	0.265	18.26	1	<0.001	3.10 (1.85–5.21)
Sex							1.07	0.290	13.7	1	<0.001	2.93 (1.66–5.17)
Age							−0.035	0.013	7.56	1	0.006	0.965 (0.941–0.990)
Ethnicity							0.064	0.298	0.047	1	0.829	1.07 (0.595–1.91)
Model	χ^2^(1) = 13.20, *p* < 0.001				Nagelkerke R^2^ = 4.1%		χ^2^(4) = 37.21, *p* < 0.001				Nagelkerke R^2 =^ 11.4%	
GJH as defined by the 5PQ^b^												
ASD	0.450	0.181	6.18	1	0.013	1.57 (1.10–2.24)	0.568	0.187	9.23	1	0.002	1.77 (1.22–2.55)
Sex							0.833	0.182	21.0	1	<0.001	2.30 (1.61–3.28)
Age							−0.007	0.007	0.860	1	0.354	0.993 (0.979–1.01)
Ethnicity							0.170	0.205	0.688	1	0.407	1.19 (0.793–1.77)
Model	χ^2^(1) = 6.13, *p* = 0.013				Nagelkerke R^2^ = 1.4%		χ^2^(4) = 29.67, *p* < 0.001				Nagelkerke R^2^ = 6.6%	
Symptomatic^c^ GJH–BSS												
ASD	1.35	0.304	19.7	1	<0.001	3.85 (2.12–6.99)	1.58	0.316	25.0	1	<0.001	4.86 (2.62–9.03)
Sex							1.48	0.375	15.5	1	<0.001	4.38 (2.10–9.14)
Age							−0.025	0.014	3.01	1	0.083	0.975 (0.948–1.00)
Ethnicity							0.073	0.355	0.043	1	0.837	1.08 (0.536–2.16)
Model	χ^2^(1) = 20.10, *p* < 0.001				Nagelkerke R^2^ = 7.6%		χ^2^(4) = 42.39, *p* < 0.001				Nagelkerke R^2^ = 15.7%	
Symptomatic GJH−5PQ												
ASD	0.776	0.197	15.5	1	<0.001	2.17 (1.48–3.20)	0.903	0.204	19.5	1	<0.001	2.47 (1.65–3.68)
Sex							0.914	0.209	19.2	1	<0.001	2.49 (1.66–3.75)
Age							0.004	0.008	0.224	1	0.636	1.00 (0.988–1.02)
Ethnicity							0.217	0.227	0.912	1	0.339	1.24 (0.796–1.94)
Model	χ^2^(1) = 15.30, *p* < 0.001				Nagelkerke R^2^ = 3.7%		χ^2^(4) = 36.90, *p* < 0.001				Nagelkerke R^2^ = 8.9%	

*Abbreviations: 5PQ, five–part questionnaire on hypermobility; ASD, autism spectrum disorder; BSS, Beighton scoring system; CI, confidence interval; GJH, generalised joint hypermobility; OR, odds ratio*.

*Note: Adjusted models have been controlled for sex, age, and ethnicity. ASD is for ASD diagnosis compared to no ASD diagnosis. Sex is for women compared to men. Ethnicity is for one or both parents born outside of the Nordic countries compared to no parent born outside of the Nordic countries. All p values are 2–sided*.

a *GJH as defined by the Beighton scoring system; age–dependent cut–off score of ≥ 5/9 for individuals 18–50 years and ≥4/9 for individuals > 50 years*.

b *GJH as defined by the 5PQ; cut–off score ≥ 2/5*.

c *Symptomatic GJH–BSS and symptomatic GJH−5PQ were defined as GJH (as defined by the BSS and the 5PQ, respectively) combined with ≥1 out of 4 self–reported items: (1) back or joint pain, (2) dislocation of shoulder or patella more than once as a child or teenager, (3) skin hyperelasticity or (4) velvety skin*.

### Subgroup Analyses on ASD Without Comorbid ADHD

Sixty-one of the 199 participants with ASD reported no occurrence of a lifetime ADHD diagnosis (Table 1). Additional exclusions depended on the specific analyses (Supplementary Table S1); for the BSS analyses, 60 participants with ASD without comorbid ADHD and 136 participants with ASD with comorbid ADHD were included for GJH, while 55 participants with ASD without comorbid ADHD and 128 participants with ASD with comorbid ADHD were included for symptomatic GJH. For the 5PQ analyses, 57 participants with ASD without comorbid ADHD and 134 participants with ASD with comorbid ADHD were included for GJH, while 56 participants with ASD without comorbid ADHD and 129 participants with ASD with comorbid ADHD were included for symptomatic GJH. The results of the four subgroup logistic regression analyses performed are presented in Table 4. Sex-stratified comparisons of prevalence rates of GJH between ASD and non-ASD controls are presented in Table 5 and comparisons of characteristics between ASD patients with and without comorbid ADHD are presented in Supplementary Table S1.

**Table 4:** Subgroup analyses on ASD with and without comorbid ADHD: Results of the logistic regression models on ASD diagnosis relationship with generalised joint hypermobility.

	**Unadjusted models**						**Adjusted models**					
**Predictor**	**B**	**SE**	**Wald**	**df**	***p***	**Unadjusted OR (95% CI)**	**B**	**SE**	**Wald**	**df**	***p***	**Adjusted OR (95% CI)**
**ASD WITHOUT COMORBID ADHD SUBGROUP**												
GJH as defined by the BSS^a^												
ASD	0.516	0.419	1.52	1	0.218	1.68 (0.737–3.81)	0.653	0.429	2.31	1	0.128	1.92 (0.828–4.46)
Sex							0.979	0.376	6.76	1	0.009	2.66 (1.27–5.57)
Age							−0.027	0.016	2.91	1	0.088	0.974 (0.944–1.00)
Ethnicity							0.209	0.361	0.336	1	0.562	1.23 (0.608–2.50)
Model	χ^2^(1) = 1.39, *p* = 0.238				Nagelkerke R^2^ = 0,6%		χ^2^(4) = 13.21, *p* = 0.010				Nagelkerke R^2^ = 6.0%	
GJH as defined by the 5PQ^b^												
ASD	0.026	0.304	0.007	1	0.933	1.03 (0.566–1.86)	0.093	0.312	0.089	1	0.766	1.10 (0.595–2.02)
Sex							0.809	0.213	14.4	1	<0.001	2.25 (1.45–3.41)
Age							−0.010	0.008	1.53	1	0.217	0.990 (0.973–1.01)
Ethnicity							0.374	0.231	2.62	1	0.105	1.45 (0.924–2.29)
Model	χ^2^(1) = 0.007, *p* = 0.933				Nagelkerke R^2^ = 0.0%		χ^2^(4) = 20.14, *p* < 0.001				Nagelkerke R^2^ = 5.8%	
Symptomatic^c^ GJH–BSS												
ASD	1.21	0.446	7.33	1	0.007	3.35 (1.40–8.02)	1.35	0.460	8.60	1	0.003	3.85 (1.56–9.47)
Sex							1.31	0.508	6.61	1	0.010	3.69 (1.36–9.99)
Age							−0.021	0.019	1.24	1	0.265	0.979 (0.944–1.02)
Ethnicity							0.094	0.450	0.044	1	0.834	1.10 (0.455–2.66)
Model	χ^2^(1) = 6.26, *p* = 0.012				Nagelkerke R^2^ = 3.6%		χ^2^(4) = 16.30, *p* = 0.003				Nagelkerke R^2^ = 9.4%	
Symptomatic GJH−5PQ												
ASD	0.384	0.326	1.39	1	0.239	1.47 (0.775–2.78)	0.448	0.333	1.81	1	0.179	1.57 (0.814–3.01)
Sex							0.818	0.251	10.66	1	0.001	2.27 (1.34–3.70)
Age							0.000	0.010	0.003	1	0.958	1.00 (0.981–1.02)
Ethnicity							0.454	0.258	3.11	1	0.078	1.56 (0.951–2.61)
Model	χ^2^(1) = 1.33, *p* = 0.249				Nagelkerke R^2^ = 0.4%		χ^2^(4) = 16.14, *p* = 0.003				Nagelkerke R^2^ = 5.3%	
**ASD WITH COMORBID ADHD SUBGROUP**												
GJH as defined by the BSS^a^
ASD	1.08	0.274	15.6	1	<0.001	2.95 (1.73–5.05)	1.31	0.289	20.6	1	<0.001	3.71 (0.2.10–6.53)
Sex							1.10	0.311	12.5	1	<0.001	3.00 (1.63–5.52)
Age							−0.038	0.014	7.74	1	0.005	0.963 (0.937–0.989)
Ethnicity							0.132	0.315	0.174	1	0.676	1.14 (0.615–2.12)
Model	χ^2^(1) = 14.88, *p* < 0.001				Nagelkerke R^2^ = 5.2%		χ^2^(4) = 37.39, *p* < 0.001				Nagelkerke R^2^ = 12.8%	
GJH as defined by the 5PQ^b^												
ASD	0.619	0.203	9.30	1	0.002	1.86 (1.25–2.77)	0.772	0.212	13.3	1	<0.001	2.17 (1.43–3.28)
Sex							0.896	0.193	21.4	1	<0.001	2.45 (1.68–3.56)
Age							−0.008	0.008	1.01	1	0.314	0.992 (0.978–1.01)
Ethnicity							0.135	0.216	0.389	1	0.533	1.15 (0.749–1.75)
Model	χ^2^(1) = 9.20, *p* = 0.002				Nagelkerke R^2^ = 2.3%		χ^2^(4) = 32.91, *p* < 0.001				Nagelkerke R^2^ = 8.0%	
Symptomatic^c^ GJH–BSS												
ASD	1.41	0.328	18.4	1	<0.001	4.08 (2.15–7.75)	1.70	0.347	24.0	1	<0.001	5.47 (2.77–10.8)
Sex							1.60	0.420	14.5	1	<0.001	4.97 (2.18–11.3)
Age							−0.0.28	0.016	3.22	1	0.073	0.972 (0.943–1.00)
Ethnicity							0.152	0.385	0.156	1	0.693	1.16 (0.547–2.48)
Model	χ^2^(1) = 17.75, *p* < 0.001				Nagelkerke R^2^ = 7.7%		χ^2^(4) = 38.94, *p* < 0.001				Nagelkerke R^2^ = 16.5%	
SYMPTOMATIC GJH−5PQ												
ASD	0.932	0.218	18.2	1	<0.001	2.54 (1.66–3.90)	1.09	0.229	22.7	1	<0.001	2.97 (1.90–4.65)
Sex							0.941	0.223	17.8	1	<0.001	2.56 (1.66–3.97)
Age							0.004	0.008	0.268	1	0.605	1.00 (0.988–1.02)
Ethnicity							0.190	0.241	0.619	1	0.432	1.21 (0.753–1.94)
Model	χ^2^(1) = 17.66, *p* < 0.001				Nagelkerke R^2^ = 4.8%		χ^2^(4) = 37.83, *p* < 0.001				Nagelkerke R^2^ = 10.0%	

*Abbreviations: 5PQ, five–part questionnaire on hypermobility; ADHD, attention–deficit/hyperactivity disorder; ASD, autism spectrum disorder; BSS, Beighton scoring system; CI, confidence interval; GJH, generalised joint hypermobility; OR, odds ratio*.

*Note: Subgroup analyses on ASD with and without comorbid ADHD. Adjusted models have been controlled for sex, age, and ethnicity. ASD is for ASD diagnosis compared to no ASD diagnosis. Sex is for women compared to men. Ethnicity is for one or both parents born outside of the Nordic countries compared to no parent born outside of the Nordic countries. All p values are 2-sided*.

a *GJH as defined by the Beighton scoring system; age–dependent cut–off score of ≥ 5/9 for individuals 18–50 years and ≥4/9 for individuals > 50 years*.

b *GJH as defined by the 5PQ; cut–off score ≥ 2/5*.

c *Symptomatic GJH–BSS and symptomatic GJH−5PQ were defined as GJH (as defined by the BSS and the 5PQ, respectively) combined with ≥1 out of 4 self–reported items: (1) back or joint pain, (2) dislocation of shoulder or patella more than once as a child or teenager, (3) skin hyperelasticity, or (4) velvety skin*.

**Table 5:** Subgroup analyses on ASD with and without comorbid ADHD: Prevalence of generalized joint hypermobility and symptomatic generalized joint hypermobility.

	**Group affiliation**		**Chi–Square tests**		**Risk estimate**	
	**ASD**	**Non–ASD**	**χ2** **(***df***=1)**	**p**	**OR**	**(95% CI)**
**ASD WITHOUT COMORBID ADHD SUBGROUP**						
GJH as defined by the BSS^a^, *n* (%)					
Women	6 (20.0)	27 (11.0)	Fisher's	0.146	2.03	(0.761–5.40)
Men	2 (6.7)	8 (4.7)	Fisher's	0.648	1.45	(0.292–7.17)
GJH as defined by the 5PQ^b^, *n* (%)				
Women	11 (36.7)	94 (38.2)	0.027	0.869	0.936	(0.427–2.05)
Men	7 (25.9)	36 (20.8)	0.362	0.547	1.33	(0.523–3.40)
Symptomatic^c^ GJH–BSS, *n* (%)					
Women	6 (20.7)	17 (7.0)	Fisher's	0.024	3.48	(1.25–9.71)
Men	2 (7.7)	3 (1.8)	Fisher's	0.133	4.61	(0.733–29.0)
Symptomatic GJH−5PQ, *n* (%)					
Women	10 (34.5)	61 (25.0)	1.21	0.271	1.58	(0.696–3.58)
Men	5 (18.5)	22 (12.8)	Fisher's	0.379	1.55	(0.532–4.51)
**ASD WITH COMORBID ADHD SUBGROUP**						
GJH as defined by the BSS^a^, *n* (%)						
Women	20 (31.7)	27 (11.0)	16.8	<0.001	3.77	(1.94–7.33)
Men	9 (12.3)	8 (4.7)	4.56	0.033	2.85	(1.05–7.71)
GJH as defined by the 5PQ^b^, *n* (%)					
Women	36 (58.1)	94 (38.2)	8.00	0.005	2.24	(1.27–3.94)
Men	25 (34.7)	36 (20.8)	5.26	0.022	2.02	(1.10–3.72)
Symptomatic^c^ GJH–BSS, *n* (%)					
Women	17 (28.8)	17 (7.0)	22.8	<0.001	5.41	(2.56–11.4)
Men	5 (7.2)	3 (1.8)	Fisher's	0.048	4.32	(1.00–18.6)
Symptomatic GJH−5PQ, *n* (%)					
Women	32 (53.3)	61 (25.0)	18.2	<0.001	3.43	(1.91–6.15)
Men	18 (26.1)	22 (12.8)	6.29	0.012	2.41	(1.20–4.84)

*Abbreviations: 5PQ, five–part questionnaire on hypermobility; ADHD, attention–deficit/hyperactivity disorder; BSS, Beighton scoring system; GJH, generalised joint hypermobility disorder; OR, odds ratio*.

*Note: Subgroup analyses on ASD with and without comorbid ADHD. Comparisons are made between participants with ASD and non–ASD controls. Difference between groups were analysed by Pearson Chi–square test*.

*Fisher's exact test was used when expected value of a cell was < 5. All p values are 2–sided*.

a *GJH as defined by the Beighton scoring system; age–dependent cut–off score of ≥ 5/9 for individuals 18–50 years and ≥4/9 for individuals > 50 years*.

b *GJH as defined by the 5PQ; cut–off score ≥ 2/5*.

c *Symptomatic GJH–BSS and symptomatic GJH−5PQ were defined as GJH (as defined by the BSS and the 5PQ, respectively) combined with ≥1 out of 4 self–reported items: (1) back or joint pain, (2) dislocation of shoulder or patella more than once as a child or teenager, (3) skin hyperelasticity or (4) velvety skin*.

## Discussion

The present study is to date by far the largest study to evaluate the association between ASD and GJH and to measure GJH in all study participants. We present novel findings regarding the relationship between GJH and ASD in adults. GJH was assessed by self-report and a physical examination in a large sample of psychiatric patients with ASD and in non-ASD community controls. These findings indicate that GJH and ASD are related in adults. To the best of our knowledge, the current study was the first to evaluate the relationship between GJH and ASD in adults, using a case-control design and assessing joint hypermobility status in all study participants.

### ASD *per se* vs. ASD Comorbid With ADHD

Our results raise two important questions; (1) Is the association between ASD and GJH less prominent than for ADHD and GJH? and (2) Is the association between ASD and GJH mainly driven by an ADHD phenotype?

The considerable overlap between these two diagnoses has been recognised for over a decade (3, 34). Young children with autism are often hyperactive and inattention is similarly common across these diagnoses, which is why clinicians have been inclined to view these symptoms in patients with ASD as features of the ASD. However, impulsivity, emotional dysregulation, risk taking behaviours, and conduct problems, all symptoms associated with ADHD, have not been as commonly reported in ASD, motivating clinicians to add a comorbid ADHD diagnosis in cases with these features. This has continuously increased since the advent of the DSM-5, which allows for this comorbidity. In line with this, we considered ASD as the primary diagnosis and ADHD as a comorbidity.

Individuals with ASD without any other co-existing psychiatric disorder are in clear minority (5, 6). DSM-5 subtypes ASD according to if the condition is: (a) with or without accompanying intellectual impairment, (b) with or without accompanying language impairment (c) associated with a known medical or genetic condition or environmental exposure, (d) associated with another neurodevelopmental, mental or behavioural disorder and (e) associated with catatonia (1). Thus, there are numerous different parameters on which a subclassification can be based. Our sample size was calculated to allow adjusted analyses for potential confounding effects of age, sex and ethnicity. However, we did not include any subgroup analyses in the sample size estimations. Thus, the subgroup analyses on ASD with and without comorbid ADHD should be viewed as *post-hoc* analyses to generate hypotheses for future studies.

In the current study sample, the logistic regression models revealed a significant association between ASD and GJH and between ASD and symptomatic GJH, as assessed by the BSS, with adjusted odds ratios of 3.6 (95% CI: 2.1, 6.2, *p* < 0.001) and 5.4 (95% CI: 2.8, 10.5, *p* < 0.001), respectively (Table 3). This is slightly lower than what was found for GJH and adult ADHD without comorbid ASD (26) using the same control group and methodology as the present study. Although the putative difference in strengths of two the associations has not been systematically evaluated, these findings indicate that the association between ASD and GJH is less pronounced than the association between ADHD and GJH. Hypothetically, there may be several aetiopathologically distinct GJH subgroups (representing various GJH genocopies as well as phenocopies) within the psychiatric disorders. Therefore, one possible explanation to what appears to be a stronger link between GJH and ADHD than between GJH and ASD is that there are fewer and/or smaller subgroups with a GJH phenotype within the ASD population than within the ADHD population.

In the present study, subgroup analyses were conducted on participants with ASD without comorbid ADHD (*n* = 61). In these analyses, a significant association between ASD and GJH only emerged in the regression model with symptomatic GJH (Table 4). However, these subgroup analyses were not included in the sample size calculations, therefore, type II error cannot be ruled out. Considering the extensive aetiological and phenotypical overlap between ASD and ADHD, and the high prevalence of a co-existing ADHD within the ASD population we argue that the present study supports the association between ASD and GJH in adults. Yet, it remains unresolved if an ADHD phenotype is the main driving force behind this association. In this cohort, 69% of the participants with ASD were also diagnosed with ADHD. This remarkably high comorbidity rate could be related to the sample selection. Participants with ASD were recruited only from psychiatric clinics attended by individuals requiring medical measures, e.g., medication and other psychiatric treatments, thereby increasing the likelihood of comorbidities. Moreover, the current study did not include any known genetic forms of ASD. Whilst genetic syndromes are rare in ASD (only found in 5% of the total ASD population (35), many syndromic forms of ASD share a hypermobile phenotype (e.g., Fragile X syndrome) (14, 36). The current sample was overall high functioning and, therefore, the group of individuals with early identified autism and intellectual disabilities, which tend to be associated with genetic syndromes, were missed. If we instead had selected participants from such recruitment centres as habilitative clinics, the ASD group might have presented with a different GJH pattern. In view of the heterogeneous nature of ASD (and ADHD), forthcoming studies on ASD subtypes, such as ASD without comorbid ADHD, ASD with comorbid intellectual impairment, and ASD associated with a genetic syndrome or physical features are encouraged in order to improve the understanding of the association.

### GJH and Symptomatic GJH

GJH is broadly defined and is likely to include a diverse number of aetiologies and clinical presentations. In the current study, manifestations suggestive of symptomatic GJH (e.g., HSD/h-EDS) were evaluated by using four self-report questions on musculoskeletal symptoms and skin abnormalities. All four of the items were significantly more common amongst participants with ASD than amongst the controls without ASD (Table 1). As many as two-thirds of the participants with ASD reported frequent back- or joint pain. Additionally, participants with ASD had three-fold more frequent recurrent dislocation of the shoulder or kneecap than did non-ASD controls, while the experience of skin-abnormalities were more than twice as common.

The musculoskeletal symptoms and skin abnormality items were also used to create a proxy for symptomatic GJH. Analyses on symptomatic GJH demonstrated a stronger association with ASD than non-specified GJH did, with an OR of 4.9 (95% CI: 2.6, 9.0) compared to 3.1 (95% CI: 1.9, 5.2), respectively. The results indicate that signs related to HSDs and connective tissue disorders are overrepresented within people with ASD. Because of this, we advocate for clinicians to be observant on musculoskeletal problems in patients with ASD, particularly since social and communicative problems may hinder access to health care (37). If acknowledged, improvement can be obtained by physical and occupational therapy (38). In the current study, only four additional manifestations of GJH were briefly examined. However, the list of suggested joint hypermobility related comorbidities is broad (e.g., functional gastrointestinal disorders, orthostatic dysfunction, pelvic prolapses), and reports of these comorbidities are increasing (10). Accordingly, more comprehensive studies are necessary to fully explore the occurrence, and consequences, of these manifestations within ASD.

Broad diagnostic criteria, overlapping symptoms, and lack of knowledge on causative mechanisms complicate the search for robust associations between conditions. The stricter criteria for hEDS, introduced in 2017, aimed to reduce the heterogeneity of the hEDS population (10). However, no underlying genes for hEDS have been identified and it is uncertain whether hEDS is a well-defined entity or just the extreme of a spectrum that ranges from “asymptomatic” GJH to HSDs and hEDS. While our pragmatically approximated subcategory of symptomatic GJH may have identified a more homogenous group in terms of clinical presentation and possibly aetiopathology, we still hold it unlikely that hEDS alone would account for the relationship between ASD and GJH found in the present study. Therefore, we encourage future research to involve both analyses of homogenic subgroups with GJH (e.g., hEDS) as well as the exploration of potential subtypes across the full GJH spectra, beyond the somewhat arbitrary diagnostic boundaries.

### Recognised Influencers of GJH

The current study was adequately powered to adjust for recognised influencers of GJH (sex, age, and country of origin) in the logistic regression models. However, sex-stratified prevalence comparisons of GJH were not included in our sample size calculations. The positive relationship of being female on GJH is well-established (11, 39, 40) and was additionally confirmed in our regression models (Tables 3, 4). When the prevalence rates of GJH were compared between patients with ASD and non-ASD controls, no significant difference emerged for males when GJH was defined by the BSS (Table 2). This may be due to the low number of males in the current study meeting the age-dependent criteria for GJH on the BSS. Indeed, additional analyses with a cut-off ≥ 4/9, applied to all participants, regardless of age, yielded a significant difference for males. Adjustments to the cut-off value not only for age but also for sex (and country of origin), has been suggested, but not yet agreed upon (39, 40). Given the strong influence of sex on GJH, sample sizes that enable sex-specific analyses seem warranted. An improved understanding of sex differences may contribute to the understanding of the biological mechanisms contributing to these conditions and to the identification of potential sex-specific profiles.

Age has revealed a negative effect on GJH (11). One Australian study reported that the likelihood of being categorised as GJH (BSS ≥ 4/9) declined at 5.5% for each year of increasing age (39). To adjust for the potentially confounding effect of age beyond the considerations in the BSS and the 5PQ, age (in years) was included as a covariate in the logistic regression models. In a sensitivity analysis without age as an independent variable, the effect of GJH on ASD remained (Supplementary Table S2).

Higher prevalence rates of GJH are reported for Arab (40, 41), Asian (40, 42), and African (40, 43) populations compared to Caucasian populations (40). In our regression models, country of origin had no significant influence on GJH, which was unanticipated. A sensitivity analyses with more detailed information on origin confirmed the lack of significant influence in the current sample (Supplementary Table S3).

Last, the robustness of the current findings was additionally assessed by including the variable “any anxiety disorder” as a variable in the regression models. To date, anxiety is the primary psychiatric condition that has revealed the most solid association with GJH (44). In the current sample, anxiety had no significant effect on GJH in any of the regression models, while the association between ASD and GJH remained (Supplementary Table S4). This supports an association between ASD and GJH independent of anxiety. However, the present study was not designed to assess the relationship between anxiety and GJH. Apart from constituting a number of specific anxiety diagnoses, anxiety is present in almost every psychiatric disorder. Therefore the effect of anxiety may be attenuated by the vast comorbidity among ASD participants. Furthermore, participants were asked if they had been diagnosed with any anxiety disorder, not if they were suffering from anxiety. Symptoms of anxiety could have a different association to GJH than a clinical diagnosis, but this was not fully examined in the present study.

### The Use of Two Independent Assessment Methods for GJH

To control for the risk of observer bias and to facilitate the comparability of the results, two independent assessment methods were used for GJH. Consistent with expectations, the relationship between ASD and GJH was stronger for GJH as defined by the BSS than for GJH as defined by the 5PQ. The 5PQ originally was developed as a screening tool (32) and is less specific than a physical examination. That being said, the 5PQ may capture aspects of GJH overlooked by the BSS, which examines the presence of hypermobility in five joints only and is weighted towards joints in the upper limbs. The 5PQ is available in English (32), Portuguese (45), and Swedish (33) and its accessibility lends itself to large population-based surveys.

### The Prevalence Rates of GJH in Our Control Participants

The estimated population prevalence rates of GJH vary extensively between studies (11, 40). In the current control sample, the prevalence rates of GJH according to the BSS were 11% for females and 4.7% for males. A recent study using university students in North America revealed a similar, but higher prevalence of GJH, at 15.4% for females and 8.4% for males (46). The lower prevalence rates of GJH in this sample may be related to the assessment or to the age and racial characteristics of the cohort.

### The Psychiatric Characteristics of Our Study Sample

In the current study sample, the three most common additional psychiatric disorders amongst patients with ASD were depression, ADHD, and any anxiety disorder (Table 1). This is consistent with reports from a recent meta-analysis on adults with ASD (6). However, the prevalence rates were higher in the current sample as compared to the meta-analysis. The higher prevalence rates in this study may be related to the recruitment methods and that lifetime occurrence of psychiatric diagnoses was assessed. Higher morbidity burden is associated with more frequent visits to the clinic, thereby increasing the likelihood of study participation. Furthermore, the majority of cases were recruited from a combined ASD and ADHD outpatient clinic for patients requiring contact with a physician. Consequently, individuals with ASD without such needs (e.g., prescription of drugs, doctors' notes, etc.) were not likely to be recruited. Amongst controls, the prevalence rates of psychiatric diagnoses were similar or slightly lower than those reported elsewhere (47, 48). The lower estimates may be related to the relatively young age of our control participants and the eligibility criteria applied for each study.

### GJH as a Biomarker in Psychiatry

The current study adds to the growing body of literature on links between GJH and psychiatric conditions (49, 50). To date, the most solid association is for GJH and anxiety (44), while the evidence for GJH and ADHD is increasing (26, 51, 52). There are also preliminary links between GJH and obsessive-compulsive personality disorder, developmental coordination disorder, and depression (49, 50). It is plausible that GJH could serve as a biomarker to subtype psychiatric patients following the concept of precision psychiatry and function as a prognostic factor. Precision psychiatry attempts to optimise patient care by giving a more accurate biologically based diagnosis and by employing tailored interventions based on those diagnoses. Bulbena et al. have proposed the “neuroconnective phenotype” as a model of illness for anxiety with comorbid hEDS (44). They describe associated features within five dimensions (behavioural, psychopathology, somatic symptoms, somatosensory symptoms, and somatic illnesses) that can guide the clinician to comprehensive patient care (53). A similar approach could be adopted to patients with ASD with comorbid GJH, with the addition of physical features (e.g., marfanoid or androgyne features or minimal physical abnormalities). Androgynous facial features was demonstrated to be strongly and positively correlated with autistic traits as measured by the Autism-Spectrum Quotient (54). Moreover, many of the specifications within the aforementioned somatic symptom dimensions (e.g., irritable bowel syndrome, chronic widespread pain, fibromyalgia, chronic fatigue syndrome, and dysautonomia) may mimic or aggravate psychiatric symptoms such as depression, exhaustion, anxiety, and/or medication side effects, which ideally should be targeted in future studies.

Biomarkers may also help clarify the aetiology and pathogenesis of a disorder. To date, little is known about the causes of ASD (1) or GJH (10, 12). Consequently, possible mechanisms responsible for their association have yet to be elucidated. In a simplified theory, the two conditions may either be a consequence of each other (i.e. GJH causes ASD or ASD causes GJH) or share a common causative process. Considering that both conditions often present in early childhood (1, 10), the opinion of the authors is that a shared pathogenic process is likely. Exploring the pleiotropy of genes involved in syndromic forms of ASD may be helpful. Plausibly, a shared mechanism concurrently affects the development and function of the central nervous system as well as the connective tissues. Additional suggested factors contributing to the association, and to the high degree of symptom overlap between GJH and neurodevelopmental disorders, include coordination problems and sensory issues, autonomic dysfunction, immune dysregulation and environmental factors (e.g. psychosocial stressors and stigma) (14, 55).

The results of the present study indicate an augmenting role of symptomatic GJH and an ADHD phenotype on the association between ASD and GJH. Baeza-Velasco et al. have hypothesised how recognised features of GJH may promote an ADHD phenotype (56, 57). However, significantly more research is required for an evidence-based understanding on how, and to what extent, various underlying mechanisms may contribute to the association between GJH and neurodevelopmental disorders.

#### Study Strengths and Limitations

The present study has a number of strengths. First, unlike registry-based studies, joint hypermobility status was measured in all study participants. Second, two different assessment tools were used to assess GJH. Third, the large study sample enabled us to adjust for covariates that could potentially confound the association. Fourth, participants were recruited from psychiatric clinics that specialise in adult ASD and ADHD, which secures a high diagnostic correctness among participants as compared to studies where participants are recruited through websites and self-report their ASD diagnosis.

The results of the current study also have to be interpreted in light of its limitations. The cross-sectional study design prevents conclusions about any temporal aspects of the association. The ASD sample was primarily selected through out-patient psychiatric clinics; using a different source for recruitment may have resulted in a different association between ASD and GJH. Furthermore, the convenience sampling method may limit the generalizability of our results. There is also a risk of self-selection bias since hypermobile individuals may be more motivated to participate. Yet, the oral information given to eligible participants encouraged everyone to participate, regardless of self-perceived joint mobility. Additionally, compensation for participation consisted of a small box of chocolate, which should reduce selection bias (58). Last, both cases and controls are likely to be equally affected by self-selection bias. Sex-stratified prevalence comparisons of GJH and subgroup analyses on patients with ASD without comorbid ADHD were not accounted for in the sample size calculations. Additionally, data on psychiatric diagnoses relied on self-report and did not include information about when or where the person had received the diagnoses. A proxy for ethnicity was used (country of origin). Finally, there are several limitations with our analyses on symptomatic GJH, including that the conventional criteria to identify HSD/hEDS was not followed, the selected items suggestive of HSD/hEDS may also have captured recognised symptoms within the ASD spectrum (e.g., motor difficulties and sensory hypersensitivity), we did not physically examine the reported skin abnormalities, and the severity of pain or how it affected daily function was not recorded. Therefore, the interpretation of these findings should be taken with caution.

## Conclusion

The current study demonstrates that ASD and GJH are related conditions in adults. However, the high prevalence of comorbid ADHD in the current study sample reduces the generalizability of the results in patients with ASD without comorbid ADHD, which highlights the importance of subclassifications. Considering the extensive aetiological and phenotypical overlap between ASD and ADHD, and the high prevalence of a co-existing ADHD within the ASD population we argue that the present study supports the association between ASD and GJH in adults. Yet, it remains unresolved if an ADHD phenotype is the main driving force behind this association. Additionally, GJH with additional musculoskeletal- and skin symptoms demonstrated a stronger association with ASD than did non-specified GJH, indicating that symptomatic GJH may play a greater role in the relationship than non-specified GJH does.

Identifying GJH subgroup(s) within psychiatry may also have several implications. First, recognising the burdens related to GJH would allow for an earlier diagnosis and treatment. Second, symptoms related to GJH such as pain, fatigue, and orthostatic intolerance may mimic psychiatric symptoms (e.g., depression, exhaustion, anxiety, and somatoform disorder) and distort the clinical assessment. Third, identifying comorbid conditions may provide clues to underlying aetiological and pathophysiological mechanisms. It is possible that common factors are involved in development and function of the connective tissues and the central nervous system, contributing to the association between GJH and neurodevelopmental disorders.

## Data Availability Statement

The raw data supporting the conclusions of this article will be made available by the authors, without undue reservation.

## Ethics Statement

The studies involving human participants were reviewed and approved by the Regional Ethics Review Board of Stockholm (approval numbers 2014/1742-31, 2017/1688-31, and 2017/2140-32). The patients/participants provided their written informed consent to participate in this study.

## Author Contributions

MG, MH, ME, and SB were responsible for study conceptualisation and design. MG and NT were responsible for data collection. MG, MH, and ME conducted statistical analyses. MG drafted the manuscript. MH, ME, NT, and SB provided critical revision of the manuscript for important intellectual content. All authors approved the final version.

## Funding

This work was supported by grants from the Swedish Research Council (K2012- 62X-22130-04-6) to SB and by grants from Bror Gadelius minnesfond (2019-2020) to MG. The funders had no role in the study design, data collection and analysis, decision to publish, or preparation of the manuscript.

## Conflict of Interest

The authors declare that the research was conducted in the absence of any commercial or financial relationships that could be construed as a potential conflict of interest.

## Publisher's Note

All claims expressed in this article are solely those of the authors and do not necessarily represent those of their affiliated organizations, or those of the publisher, the editors and the reviewers. Any product that may be evaluated in this article, or claim that may be made by its manufacturer, is not guaranteed or endorsed by the publisher.
