# Supplementary material for: The Relationship Between Generalised Joint Hypermobility and Autism Spectrum Disorder in Adults: A Large, Cross-Sectional, Case Control Comparison
Source: Front Psychiatry. 2022 Feb 8;12:803334. doi: 10.3389/fpsyt.2021.803334 (PMC8861852; doi:10.3389/fpsyt.2021.803334)
Supplement: Supplementary file 1 [file Data_Sheet_1.zip › UPLOAD 2/S1.DOCX]

**Table S1.** Subgroup comparisons of characteristics. Comparisons are made between patients with ASD without comorbid ADHD and patients with ASD with comorbid ADHD

|  | Group affiliation | |  |
| --- | --- | --- | --- |
|  | ASD without ADHD | ASD with ADHD |  |
| Variable | N=61 | N=138 | Test of difference |
| Demographics |  |  |  |
| Female sex, n (%) | 30 (49.2) | 65 (47.1) | X^2^ = .073, p = .787 |
| Age (yrs), (mean, SD) | | |  |
| Women | 32.0 (9.1) | 33.7 (12.2) | t = -.704, p = .483 |
| Men | 36.6 (12.8) | 32.4 (11.8) | t = 1.61, p = .111 |
| Ethnicity Nordic^a^, n (%) | | |  |
| Women | 22 (73.3) | 52 (80.0) | X^2^ = .530, p = .467 |
| Men | 24 (77.4) | 56 (76.7) | X^2^ = .006, p = .938 |
| Employment status, n (%) (n= 28 v. 109) | | | X^2^=1.29, p =.255 |
| Employed or Student | 10 (35.7) | 52 (47.7) |  |
| Unemployed | 18 (64.3) | 57 (52.3) |  |
| Highest completed education, n (%) (n=29 v. 112) | | | X^2^=6.74, p =.241 |
| University ≥ 3 years | 2 (6.9) | 17 (15.2) |  |
| University < 3 years | 2 (6.9) | 14 (12.5) |  |
| Upper Secondary school | 14 (48.3) | 44 (39.3) |  |
| Vocational training | 1 (3.4) | 5 (4.5) |  |
| Compulsory school | 6 (20.7) | 28 (25.0) |  |
| Unfinished compulsory school | 4 (13.8) | 4 (3.6) |  |
| Lifetime occurrence of psychiatric diagnoses^b^, n (%) | | |  |
| Depression | 44 (72.1) | 105 (76.1) | X^2^ = .352, p = .553 |
| Anxiety disorder | 18 (29.5) | 67 (48.6) | X^2^ = 6.27, p = .012 |
| Exhaustion disorder^c^ | 1 (1.6) | 12 (8.7) | F, p = .069 |
| Bipolar disorder | 4 (6.6) | 9 (6.5) | F, p = 1.000 |
| Specific learning disorder | 1 (1.6) | 11 (8.0) | F, p = .110 |
| Personality disorder | 4 (6.6) | 5 (3.6) | F, p = .460 |
| PTSD | 2 (3.3) | 6 (4.3) | F, p = 1.000 |
| Psychosis other^d^ | 3 (4.9) | 5 (3.6) | F, p = .703 |
| Eating disorder | 3 (4.9) | 1 (.7) | F, p = .087 |
| Intellectual disability | 3 (4.9) | 0 (0.0) | F, p = .028 |
| Tourette syndrome | 0 (0.0) | 2 (1.4) | F, p = 1.000 |
| Schizophrenia | 1 (1.6) | 1 (0.7) | F, p = .520 |
| Substance use disorder | 1 (1.6) | 1 (0.7) | F, p = .520 |
| Dissociative disorder | 1 (1.6) | 1 (0.7) | F, p = .520 |
| Intermittent explosive disorder | 1 (1.6) | 1 (0.7) | F, p = .520 |
| Psychiatric rating scales (mean, SD) | (n= 54 v. 124) | |  |
| ASRS total score^e^ |  |  |  |
| Women | 38.6 (13.3) | 48.0 (8.9) | t = -3.89, p <.001 |
| Men | 31.8 (8.8) | 44.0 (10.4) | t = -5.23, p <.001 |
| ASRS Hyperactivity/Impulsivity subscale | |  |  |
| Women | 16.4 (7.3) | 21.3 (6.2) | t = -3.29, p =.001 |
| Men | 12.9 (5.5) | 19.1 (6.3) | t = -4.38, p <.001 |
| ASRS Inattention subscale | |  |  |
| Women | 22.2 (7.6) | 26.7 (4.7) | t = -3.36, p =.001 |
| Men | 18.9 (5.6) | 24.9 (5.5) | t = -4.69, p <.001 |
| Autism quotient abridged 10-item version^f^ | |  |  |
| Women | 19.3 (5.3) | 19.4 (4.4) | t = -.102, p =.919 |
| Men | 16.2 (4.4) | 17.7 (4.8) | t = -1.30, p =.197 |
| Musculoskeletal symptoms and skin abnormalities^g^, n (%) (n=53 v. 117) | | | |
| Any | 40 (75.5) | 90 (76.9) | X^2^ = .043, p = .836 |
| Frequent pain in back or joints | 38 (71.7) | 78 (66.7) | X^2^ = .426, p = .514 |
| Dislocated shoulder or kneecap ≥2 | 6 (11.3) | 18 (15.4) | X^2^ = .497, p = .481 |
| Hyperelastic skin | 5 (9.4) | 13 (11.1) | X^2^ = .108, p = .742 |
| Velvety skin | 9 (17.0) | 33 (28.2) | X^2^ = 2.47, p = .116 |
| Prevalence of GJH |  |  |  |
| GJH as defined by the BSS^h^, n (%) | (60 v. 136) | |  |
| Women | 6 (20) | 20 (31.7) | X^2^ = 1.39, p =.238 |
| Men | 2 (6.7) | 9 (12.3) | F, p =.503 |
| GJH as defined by the 5PQ^i^, n (%) | (57 v. 134) |  |  |
| Women | 11 (36.7) | 36 (58.1) | X^2^ = 3.71, p =.054 |
| Men | 7 (25.9) | 25 (34.7) | X^2^ = .695, p =.405 |
| Symptomatic^j^ GJH-BSS, n (%) | (55 v. 128) |  |  |
| Women | 6 (20.7) | 17 (28.8) | X^2^ = .665, p =.415 |
| Men | 2 (7.7) | 5 (7.2) | F, P=1.000 |
| Symptomatic GJH-5PQ, n (%) | (56 v. 129) |  |  |
| Women | 10 (34.5) | 32 (53.3) | X^2^ = 2.79, p =.095 |
| Men | 5 (18.5) | 18 (26.1) | X^2^ = .610, p =.435 |

*Abbreviations:* ASD, autism spectrum disorder; ADHD, attention-deficit/hyperactivity disorder; ASRS, Adult ADHD Self Report Scale; PTSD, post-traumatic stress disorder; X^2^, Pearson Chi-squared test; t, Student t-test; F, Fisher’s exact test; GJH, generalised joint hypermobility; 5PQ, five-part questionnaire on hypermobility.

*Note:* Age analysed by Student t-test and categorical variables by Pearson Chi-squared test. Fisher’s exact test was used when expected value of a cell was < 5. All *p* values are 2-sided. Missing data on a variable served as exclusion criteria, therefore the number of included participants differ between variables.

a. Neither parent born outside of the Nordic countries.

b. Lifetime occurrence of self-reported psychiatric diagnoses. Those with a reported prevalence lower than 1% are not shown.

c. Exhaustion disorder was introduced as a medical diagnosis in Sweden by the Swedish National Board of Health and Welfare in 2010 and is equivalent to “burnout”.

d. Unspecified psychosis not due to a substance or known physiological condition (n=7), Substance-induced psychosis (n=1).

e. Adult ADHD Self Report Scale; continuous scoring method (0–4 on each item, total score range 0-72).

f. Autism quotient abridged 10-item version, continuous scoring method (0–3 on each item, total score range 0-30).

g. Symptoms suggestive of symptomatic GJH (e.g. HSDs or h-EDS), were assessed by the four items; “Do you often have pain in your back or in your joints?”; “Do you have hyperelastic skin?”; “Do you have velvety skin?”, and “As a child or teenager, did your kneecap or shoulder dislocate on more than one occasion?”.

h. GJH as defined by the Beighton scoring system: age-dependent cut-off score of ≥ 5/9 for individuals 18-50 years and ≥4/9 for individuals > 50 years.

i. GJH as defined by the 5PQ: cut-off score ≥ 2/5.

j. Symptomatic GJH-BSS and symptomatic GJH-5PQ were defined as GJH (as defined by the BSS and the 5PQ, respectively) combined with ≥1 out of 4 self-reported items: 1) back or joint pain, 2) dislocation of shoulder or patella more than once as a child or teenager, 3) skin hyperelasticity or 4) velvety skin.
