# Supplementary material for: The Relationship Between Generalised Joint Hypermobility and Autism Spectrum Disorder in Adults: A Large, Cross-Sectional, Case Control Comparison
Source: Front Psychiatry. 2022 Feb 8;12:803334. doi: 10.3389/fpsyt.2021.803334 (PMC8861852; doi:10.3389/fpsyt.2021.803334)
Supplement: Supplementary file 1 [file Data_Sheet_1.zip › UPLOAD 2/S2.DOCX]

**Table S2.** Results of the logistic regression models on ASD diagnosis relationship with generalised joint hypermobility. Sensitivity analysis without age as an independent variable in the models

|  | B | SE | Wald | *df* | P | Adjusted model  OR (95% CI) |
| --- | --- | --- | --- | --- | --- | --- |
| Predictor | | | | | | |
| GJH as defined by the BSS^a^ | | | | | | |
| ASD | 1.08 | .261 | 16.9 | 1 | < .001 | 2.93 (1.76-4.89) |
| Sex | 1.06 | .288 | 13.42 | 1 | < .001 | 2.88 (1.63-5.06) |
| Ethnicity | .183 | .294 | .388 | 1 | .533 | 1.2 (.675-2.14) |
| Model | χ^2^(3) = 28.56, p < .001 | | | | Nagelkerke R^2^ = 8.8% | |
| GJH as defined by the 5PQ^b^ | | | | | | |
| ASD | .560 | .187 | 8.99 | 1 | .003 | 1.75 (1.21-2.52) |
| Sex | .829 | .182 | 20.8 | 1 | < .001 | 2.29 (1.61-3.27) |
| Ethnicity | .197 | .203 | .944 | 1 | .331 | 1.22 (.818-1.81) |
| Model | χ^2^(3) = 28.80 p < .001 | | | | Nagelkerke R^2^ = 6.4% | |
| Symptomatic^c^ GJH-BSS | | | | | | |
| ASD | 1.54 | .314 | 24.2 | 1 | < .001 | 4.68 (2.53-8.65) |
| Sex | 1.47 | .374 | 15.4 | 1 | < .001 | 4.34 (2.09-9.04) |
| Ethnicity | .156 | .352 | .196 | 1 | .658 | 1.17 (.586-2.33) |
| Model | χ^2^(3) = 39.10 p < .001 | | | | Nagelkerke R^2^ = 14.5% | |
| Symptomatic GJH-5PQ | | | | | | |
| ASD | .907 | .204 | 19.74 | 1 | < .001 | 2.48 (1.66-3.70) |
| Sex | .915 | .208 | 19.26 | 1 | < .001 | 2.50 (1.66-3.76) |
| Ethnicity | .201 | .225 | .798 | 1 | .372 | 1.22 (.787-1.90) |
| Model | χ^2^(3) = 36.67 , p < .001 | | | | Nagelkerke R^2^ = 8.8% | |

*Abbreviations:* 5PQ, the five-part questionnaire on hypermobility; ASD, autism spectrum disorder; BSS, Beighton scoring system; CI, confidence interval; GJH, generalised joint hypermobility; OR, odds ratio.

*Note:* ASD is for ASD diagnosis compared to no ASD diagnosis. Sex is for women compared to men. Ethnicity is for one or both parents born outside of the Nordic countries compared to no parent born outside of the Nordic countries. All *p* values are 2-sided.

a. GJH as defined by the Beighton scoring system; age-dependent cut-off score of ≥ 5/9 for individuals 18-50 years and ≥4/9 for individuals >50 years.

b. GJH as defined by the 5PQ; cut-off score ≥ 2/5.

c. Symptomatic GJH-BSS and symptomatic GJH-5PQ were defined as GJH (as defined by the BSS and the 5PQ, respectively) combined with ≥1 out of self-reported items: 1) back or joint pain, 2) dislocation of shoulder or kneecap more than once as a child or teenager, 3) skin hyperextensibility, or 4) velvety textured skin.
