# Supplementary material for: The Relationship Between Generalised Joint Hypermobility and Autism Spectrum Disorder in Adults: A Large, Cross-Sectional, Case Control Comparison
Source: Front Psychiatry. 2022 Feb 8;12:803334. doi: 10.3389/fpsyt.2021.803334 (PMC8861852; doi:10.3389/fpsyt.2021.803334)
Supplement: Supplementary file 1 [file Data_Sheet_1.zip › UPLOAD 2/S3.DOCX]

**Table S3.** Results of the logistic regression models on ASD diagnosis relationship with generalised joint hypermobility. Sensitivity analysis with increased details on coding for ethnicity variable

|  | B | SE | Wald | *df* | | p | Adjusted model  OR (95% CI) |
| --- | --- | --- | --- | --- | --- | --- | --- |
| Predictor | | | | | | | |
| GJH as defined by the BSS^a^ | | | | | | | |
| ASD | 1.15 | .268 | 18.6 | 1 | | <.001 | 3.17 (1.88-5.35) |
| Sex | 1.05 | .293 | 13.0 | 1 | | <.001 | 2.87 (1.62-5.09) |
| Age | -.036 | .013 | 7.68 | 1 | | .006 | .965 (.940-.990) |
| Ethnicity mother |  |  | 2.46 | 5 | | .782 |  |
| Ethnicity mother (1) | .559 | .792 | .497 | 1 | | .481 | 1.75 (.370-8.26) |
| Ethnicity mother (2) | -.735 | .895 | .674 | 1 | | .412 | .480 (.083-2.77) |
| Ethnicity mother (3) | -19.1 | 17542.0 | .000 | 1 | | .999 | .000 (.000-.) |
| Ethnicity mother (4) | 1.37 | 1.27 | 1.15 | 1 | | .283 | 3.92 (.323-47.5) |
| Ethnicity mother (5) | .311 | 1.04 | .090 | 1 | | .764 | 1.37 (.179-10.4) |
| Ethnicity father |  |  | 1.54 | 5 | | .908 |  |
| Ethnicity father (1) | -.580 | .781 | .552 | 1 | | .458 | .560 (.121-2.59) |
| Ethnicity father (2) | .804 | .867 | .860 | 1 | | .354 | 2.23 (.409-12.2) |
| Ethnicity father (3) | .132 | 1.12 | .014 | 1 | | .906 | 1.14 (.127-10.2) |
| Ethnicity father (4) | -19.5 | 211189.4 | .000 | 1 | | .999 | .000 (.000-.) |
| Ethnicity father (5) | .109 | 1.24 | .008 | 1 | | .930 | 1.12 (.099-12.6) |
| Model | χ^2^(13) = 41.92, p < .001 | | | | Nagelkerke R^2^ = 12.9% | | |
| GJH as defined by the 5PQ^b^ | | | | | | | |
| ASD | .570 | .189 | 9.10 | 1 | | .003 | 1.77 (1.22-2.56) |
| Sex | .853 | .185 | 21.4 | 1 | | <.001 | 2.35 (1.64-3.37) |
| Age | -.007 | .007 | .939 | 1 | | .332 | .993 (.979-1.01) |
| Ethnicity mother |  |  | 1.37 | 5 | | .927 |  |
| Ethnicity mother (1) | .012 | .523 | .000 | 1 | | .982 | 1.01 (.363-2.82) |
| Ethnicity mother (2) | .587 | .605 | .944 | 1 | | .331 | 1.80 (.550-5.89) |
| Ethnicity mother (3) | -.465 | 1.44 | .105 | 1 | | .746 | .628 (.038-10.5) |
| Ethnicity mother (4) | .365 | 1.10 | .109 | 1 | | .741 | 1.44 (.166-12.5) |
| Ethnicity mother (5) | -.370 | .834 | .197 | 1 | | .657 | .691 (.135-3.54) |
| Ethnicity father |  |  | 2.02 | 5 | | .846 |  |
| Ethnicity father (1) | -.108 | .474 | .052 | 1 | | .820 | .898 (.354-2.27) |
| Ethnicity father (2) | -.230 | .619 | .138 | 1 | | .710 | .794 (.236-2.67) |
| Ethnicity father (3) | -.166 | .888 | .035 | 1 | | .852 | .847 (.149-4.83) |
| Ethnicity father (4) | 1.55 | 1.30 | 1.44 | 1 | | .231 | 4.73 (.373-60.0) |
| Ethnicity father (5) | .574 | .918 | .392 | 1 | | .531 | 1.78 (.294-10.73) |
| Model | χ^2^(13) = 33.71, p = .001 | | | | Nagelkerke R^2^ = 7.5% | | |
| Symptomatic^c^ GJH-BSS | | | | | | | |
| ASD | 1.62 | .322 | 25.2 | 1 | | <.001 | 5.04 (2.68-9.47) |
| Sex | 1.46 | .38 | 14.7 | 1 | | <.001 | 4.26 (2.04-9.02) |
| Age | -.026 | .015 | 3.11 | 1 | | .078 | .974 (.947-1.00) |
| Ethnicity mother |  |  | 3.35 | 5 | | .647 |  |
| Ethnicity mother (1) | .617 | .900 | .470 | 1 | | .493 | 1.85 (.318-10.8) |
| Ethnicity mother (2) | -.306 | 1.07 | .082 | 1 | | .774 | .736 (.091-5.97) |
| Ethnicity mother (3) | -19.0 | 17425.5 | .000 | 1 | | .999 | .000 (.000-.) |
| Ethnicity mother (4) | 2.07 | 1.31 | 2.50 | 1 | | .114 | 7.89 (.609-102.1) |
| Ethnicity mother (5) | .664 | 1.13 | .345 | 1 | | .557 | 1.94 (.212-17.8) |
| Ethnicity father |  |  | .756 | 5 | | .980 |  |
| Ethnicity father (1) | -.586 | .885 | .438 | 1 | | .508 | .557 (.098-3.16) |
| Ethnicity father (2) | -.206 | 1.07 | .037 | 1 | | .847 | .814 (.100-6.63) |
| Ethnicity father (3) | .593 | 1.15 | .268 | 1 | | .605 | 1.81 (.192-17.1) |
| Ethnicity father (4) | -19.6 | 20338.0 | .000 | 1 | | .999 | .000 (.000-.) |
| Ethnicity father (5) | -.150 | 1.35 | .012 | 1 | | .911 | 1.16 (.082-16.5) |
| Model | χ^2^(13) = 48.00, p < .001 | | | | Nagelkerke R^2^ = 17.7% | | |
| Symptomatic GJH-5PQ | | | | | | | |
| ASD | .910 | .208 | 19.2 | 1 | | <.001 | 2.48 (1.65-3.73) |
| Sex | .948 | .213 | 19.8 | 1 | | <.001 | 2.58 (1.70-3.92) |
| Age | .003 | .008 | .158 | 1 | | .691 | 1.00 (.987-1.02) |
| Ethnicity mother |  |  | 3.25 | 5 | | .662 |  |
| Ethnicity mother (1) | .029 | .569 | .003 | 1 | | .959 | 1.03 (.338-3.14) |
| Ethnicity mother (2) | 1.05 | .685 | 2.35 | 1 | | .125 | 2.86 (.746-10.9) |
| Ethnicity mother (3) | .621 | 1.60 | .152 | 1 | | .697 | 1.86 (.082-42.5) |
| Ethnicity mother (4) | .970 | 1.14 | .724 | 1 | | .395 | 2.64 (.282-24.6) |
| Ethnicity mother (5) | -.086 | .929 | .009 | 1 | | .926 | .917 (.149-5.56) |
| Ethnicity father |  |  | 4.57 | 5 | | .471 |  |
| Ethnicity father (1) | .088 | .511 | .029 | 1 | | .864 | 1.09 (.401-2.97) |
| Ethnicity father (2) | -.999 | .723 | 1.91 | 1 | | .167 | .368 (.089-1.52) |
| Ethnicity father (3) | -.555 | 1.13 | .242 | 1 | | .623 | .574 (.063-5.24) |
| Ethnicity father (4) | 1.88 | 1.33 | 1.99 | 1 | | .158 | 6.55 (.482-89.1) |
| Ethnicity father (5) | .464 | 1.04 | .201 | 1 | | .654 | 1.59 (.209-12.1) |
| Model | χ^2^(13) = 43.02, p < .001 | | | | Nagelkerke R^2^ = 10.3% | | |

*Abbreviations:* 5PQ, the five-part questionnaire on hypermobility; ADHD, attention-deficit/hyperactivity disorder; BSS, Beighton scoring system; CI, confidence interval; GJH, generalised joint hypermobility; OR, odds ratio.

*Note:* ASD is for ASD diagnosis compared to no ADHD diagnosis. Sex is for women compared to men. Ethnicity mother is for mother born outside of the Nordic countries compared to mother born in the Nordic countries; (1) Europe excluding the Nordic countries (2) Asia including the Middle East (3) Africa (4) North America (5) South America including the Caribbean. Ethnicity father is for father born outside of the Nordic countries compared to mother born in the Nordic countries; (1) Europe excluding the Nordic countries (2) Asia including the Middle East (3) Africa (4) North America (5) South America including the Caribbean. All *p* values are 2-sided.

a. GJH as defined by the Beighton scoring system; age-dependent cut-off score of ≥ 5/9 for individuals 18-50 years and ≥4/9 for individuals >50 years.

b. GJH as defined by the 5PQ; cut-off score ≥ 2/5.

c. Symptomatic GJH-BSS and symptomatic GJH-5PQ were defined as GJH (as defined by the BSS and the 5PQ, respectively) combined with ≥1 out of self-reported items: 1) back or joint pain, 2) dislocation of shoulder or kneecap more than once as a child or teenager, 3) skin hyperextensibility, or 4) velvety textured skin.
